# Co-morbidity burden in Parkinson’s disease: Comparison with controls and its influence on prognosis

**DOI:** 10.1016/j.parkreldis.2016.05.013

**Published:** 2016-07

**Authors:** Angus D. Macleod, Hannah Goddard, Carl E. Counsell

**Affiliations:** a Institute of Applied Health Sciences, University of Aberdeen, Foresterhill, Aberdeen, AB25 2ZD, UK; b School of Medicine, Medical Sciences and Nutrition, University of Aberdeen, Foresterhill, Aberdeen, AB25 2ZD, UK

**Keywords:** Parkinson’s disease, Co-morbidity, Charlson, Mortality, Dependency

## Abstract

**Background:**

Many aspects of co-morbidity burden in Parkinson’s disease (PD) are unclear, but it may be an important predictor of prognosis or confounder of associations in epidemiological studies.

**Objectives:**

To determine how best to assess co-morbidity burden in PD, to compare with matched controls, and investigate its association with prognostic outcomes.

**Methods:**

Data from an incident, community-based cohort with prospective follow-up (the PINE study) were used (198 patients with PD and 151 controls). The reliability of three co-morbidity scales (the Charlson co-morbidity index (CCI), the Cumulative Illness Rating scale and a simple disease count) were evaluated. The association with mortality and development of dependency was assessed with Cox regression. The co-morbidity burden in PD and controls was compared at baseline and over 5 years of follow-up using linear mixed modelling.

**Results:**

The CCI was more reliable and was an independent predictor of mortality with a time-dependent effect (hazard ratio = 1.27 [1.08–1.49] in first four years of follow-up; no significant association after four years). Associations between the other scales and mortality and between each scale and development of dependency were non-significant once adjusted for confounders. Co-morbidity burden was similar between cases and controls at baseline and there was no evidence of differential accrual of co-morbidity between patients and controls (p = 0.94).

**Conclusions:**

The CCI is probably the better scale for measuring co-morbidity burden in PD. There were no differences between PD and controls. Co-morbidity burden at diagnosis was associated with mortality in the early part of the disease course, but not later.

## Highlights

• The Charlson Index was a reliable and valid way to measure co-morbidity burden in PD.
• There was no difference in co-morbidity burden between PD and matched controls.
• Higher co-morbidity at diagnosis was associated with higher early mortality.

## 1 Introduction

Parkinson’s disease (PD) predominantly occurs in the elderly [1], where other illnesses are common. Although certain specific co-morbid diseases have been studied in PD—for example, people with PD have a lower risk of cancer than in the general population [2]—very little is known about the overall co-morbidity burden in PD. Basic aspects of this are unclear, including whether co-morbidity burden differs from that of the general population and how best to measure it in PD. Additionally, although an increase in overall co-morbidity burden has been shown to increase mortality and disability in the general population [3], [4], its influence on prognosis in PD is unclear. In PD, co-morbidity burden may be an important independent prognostic factor and a confounder of associations or an effect modifier in epidemiological studies; thus understanding its influence on prognosis is important for studies of prognosis in PD. Measuring overall co-morbidity as a single variable, rather than multiple individual diseases, is necessary for efficiency of statistical analyses [5].

We therefore aimed to determine which scale to use to measure co-morbidity in PD; to identify whether co-morbidity influences prognosis in PD; and compare the overall co-morbidity burden in PD and controls at diagnosis and during follow-up.

## 2 Methods

### 2.1 Study design

The PINE study is an incident cohort of PD and other forms of parkinsonism with prospective life-long follow-up in North-East Scotland [6], [7]. Using multiple community-based ascertainment strategies we tried to identify all new diagnoses of degenerative or vascular parkinsonism (defined broadly as having at least two cardinal signs) during two incidence periods totaling 4.5 years (2002–2004 and 2006–2009). Patients were invited to consent to long-term follow-up. For each patient recruited, an age- and sex-matched control was sought from the same general practice. The only exclusion for patients was drug-induced parkinsonism, but controls were excluded if they were unable to give informed consent because of dementia or if they were found to be parkinsonian.

Consenting patients and controls were seen annually and underwent comprehensive clinical assessment including demographics, clinical history and examination, review of medical case notes, and both generic and PD-specific assessment scales. All participants were tagged to the NHS central register so that regular notifications of deaths were also received. Patients’ diagnoses were reviewed at each appointment by a neurologist with a special interest in movement disorders or by a supervised trainee. Diagnoses of PD were guided by the UK PD Brain Bank criteria [8]. Only those who had a diagnosis of idiopathic PD at latest follow-up, and the controls matched to these patients, were included in the analyses described here.

Ethical approval for the PINE study recruitment and follow-up was obtained from the Grampian Research Ethics Committee and the Multi-Centre Research Ethics Committee for Scotland. The participants gave written informed consent and all data were stored securely.

### 2.2 Co-morbidity data

Two sources of co-morbidity data were available: research files from the PINE study and electronic summaries of primary care records. The research files contained information about co-morbid illness gathered from participants at clinical interview at baseline (i.e. at diagnosis for patients and at recruitment for controls) and subsequent visits, and also from review of hospital case notes.

We evaluated three different scales of co-morbidity burden, two weighted scales (the Charlson co-morbidity index [CCI] [9], the Cumulative Illness Rating Scale [CIRS]) [10] and a simple unweighted disease count. The first two of these scales were chosen as they are commonly used in the epidemiological literature and have previously been used in PD [5], [11], [12], [13]. The CCI uses a specified list of mostly chronic diseases which are weighted between one and six. It has been validated in several populations and various diseases [5], [11]. The CIRS counts co-morbid diseases by body system and includes grading of severity of disease, from zero (no disease, or previous problems with no sequelae) to four (an extremely severe problem). It has been shown to be valid and reliable for use in several situations, including older populations [5], [14]. We also used an unweighted disease count as it is arguably a simpler method of evaluating co-morbidity and simple counts of disease have previously been shown to perform similarly to complex measures when predicting most outcomes, including mortality [11]. For the disease count we defined a disease as any condition requiring ongoing treatment or one that causes disordered organ function, after Gross et al. [15].

### 2.3 Comparison of co-morbidity scales and investigation of association with prognostic outcomes

We compared these three scales in PD at baseline only. Using the available data on co-morbidity, scores were calculated using each scale. The reliability of the scales was compared by assessing the intra- and inter-rater reliability with intraclass correlation coefficients [ICCs]). The first 40 cases were scored by one assessor (HG) who re-scored the first 20 of these cases 8 weeks later, blind to the first scoring. A second researcher (ADM) re-scored the other 20 cases, also blind to the first scoring.

In order to (i) assess the construct validity of the scales and (ii) to assess the effect of co-morbidity on prognosis, we investigated the association between each co-morbidity scale and two important prognostic outcomes: mortality and dependency. Dependency, (needing help with basic activities of daily living) was defined as a sustained score of <80 on the Schwab & England scale [16]. We firstly plotted Kaplan-Meier survival probabilities by categories of each co-morbidity scale. We then performed survival analysis using Cox regression using data until the end of follow-up. Separate models were created with each scale in turn as the exposure of interest (as a continuous variable), for each outcome. We generated both unadjusted models (univariable associations) and also adjusted multivariable models by adding potential confounding variables measured at diagnosis. These potential confounders were variables likely to be associated with both co-morbidity and mortality: age, sex, smoking status (ever or never), an area-based socioeconomic deprivation measure (DepCat score) [17] and severity of parkinsonian impairment (Unified Parkinson’s disease rating scale [UPDRS] motor score) [18]. The potential confounders were included in the model irrespective of their statistical significance. Smoking was not included separately in models with CIRS since it is scored within this rating scale itself. Patients lost to follow-up, or those still alive/independent at the time of data extraction from the study database were censored. Patients dependent at baseline were excluded from the models of dependency. There were no missing data for any of the baseline variables in the models.

The proportional hazards assumption was tested by formal testing based on Schoenfeld residuals [19]. If there was evidence of violation of the proportional hazards assumption for the co-morbidity scales, an interaction term between the scale and time period was added (time divided into two intervals with approximately similar numbers of events in each). A likelihood ratio test was used to compare a model with and without this interaction. Otherwise, interactions were not assessed due to lack of power. Because we found a time-varying effect of co-morbidity on mortality we reviewed the clinical data available (research records, hospital case notes, and general practice records where available) to identify whether the proportion of deaths related to PD (such as due to general frailty, pneumonia, complications of fractures, or complications of immobility) varied over time.

### 2.4 Comparison between patients and controls

For comparisons between patients and controls we used only the CCI as it demonstrated the best reliability and was the only scale to be independently associated with mortality. The distribution of baseline co-morbidity was compared between PD and controls both graphically and with the Wilcoxon rank-sum test. The effect of time on change in co-morbidity over the first five years of follow-up was assessed using a linear mixed-model to adjust for repeated measures. CCI defined the dependent variable, and time (year of follow-up) was included in the model as the co-variate of interest. An interaction between patient/control status and time was included to assess whether change in CCI varied between patients and controls. Potential confounders (age, sex, and smoking status) were included as fixed effects in the model. An autoregressive covariance structure was assumed.

Statistical analyses were performed using Stata version 12.1 (StataCorp, College Station, TX).

## 3 Results

377 incident patients with parkinsonism were identified in the incidence phase of the PINE study (see supplementary figure). Of the 355 who consented to follow-up, 198 were identified as having PD at latest follow-up. 151 age- and sex-matched controls were recruited for the PD patients (the consent rate in potential controls approached was 44%). Two cases and one control were lost to follow-up for co-morbidity between baseline assessment and the fifth year of follow-up. 57 patients and 22 controls had died by five years of follow-up. No patient was lost to follow-up for mortality data after between 6 and 12 years of follow-up). Thirty patients had sustained dependency from baseline and a further 6 patients died before their first follow-up visit so were also excluded from the analyses of survival to development of dependency so 162 were included in these analyses. Two patients were lost to follow-up for dependency data. The baseline characteristics of the cases and controls are summarized in Table 1.

### 3.1 Reliability of scales

The intra- and inter-rater reliability of the three scales are shown in Table 2. All three scales produced ICCs > 0.9 indicating excellent intra-rater reliability. The CCI had excellent inter-rater reliability with an ICC of 0.96 whereas the CIRS and the disease count had fair and moderate inter-rater reliability, respectively. The CIRS also took longer to score than the other two scales.

### 3.2 Association with prognostic outcomes

**Fig. 1:**
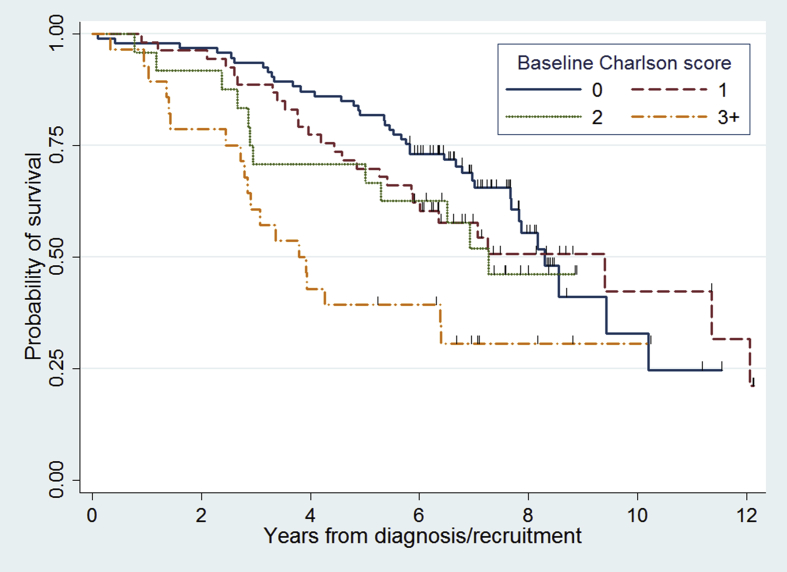
Kaplan-Meier survival probabilities in Parkinson’s disease by baseline Charlson index. Black vertical marks represent censored observations.

Hazard ratios for associations between the three scales and the outcomes are displayed in Table 2. There was strong evidence that the association between the baseline CCI and mortality varied over time (p = 0.01, test based on Schoenfeld residuals) but the proportional hazards assumption was satisfied when an interaction between time period and CCI was added, and this interaction resulted in better fit (p = 0.007). Fig. 1 shows Kaplan-Meier survival curves for different levels of the CCI. There was strong evidence that a higher baseline CCI was associated with higher mortality during the first four years of follow-up (HR 1.27 [1.08–1.49]), but there was no evidence of an association in the second period of follow-up (HR 0.84 [0.64–1.10]). There was corroborative evidence for this finding from the analysis of whether deaths were related to PD, which showed an increasing proportion of deaths related to PD over time: 15% before two years, 47% between two and four years, 58% between 4 and 6 years, and 69% between 6 and 8 years. Whilst CIRS and disease counts were associated with mortality in the univariable analyses (but with a less strong association than with the CCI) there was no evidence that they were independently predictive of mortality after adjustment for potential confounders (Table 2). Age was the key confounder leading to the apparent association in the univariable analyses. There was no robust evidence that any co-morbidity scale was associated with dependency after adjustment for confounders. Age, again, was the confounding variable which led to the stronger effect in the crude analyses.

### 3.3 Comparison between PD and controls

**Fig. 2:**
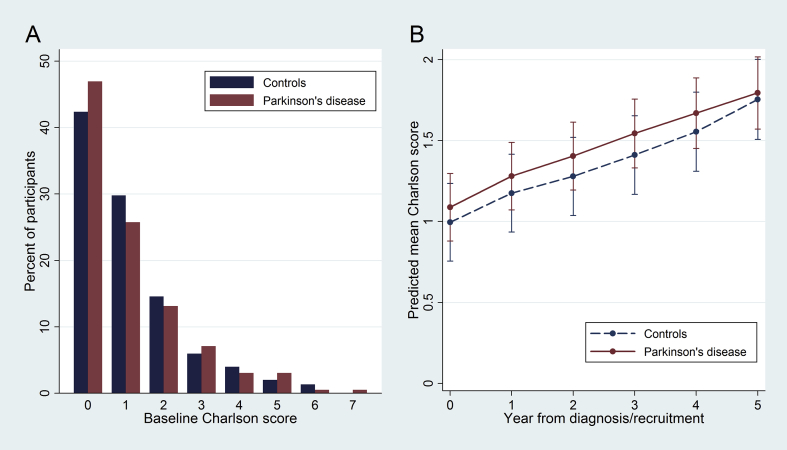
A: Distribution of baseline Charlson scores in Parkinson’s disease and in controls. B: Mean Charlson scores from linear mixed model in Parkinson’s disease and in controls over five years. Error bars represent 95% confidence intervals.

The distribution of Charlson scores in PD and controls at baseline is shown in Fig. 2A. There was no evidence of a difference between the patients and controls (p = 0.57, Wilcoxon rank-sum test). The mean increase in CCI in all participants was 0.13 points per year. Change over time in patients and controls is displayed graphically in Fig. 2B. There was no evidence that there was a different rate of accumulation of co-morbidity between patients and controls in the mixed model (p = 0.94).

## 4 Discussion

All the scales had good intra-rater reliability, but the CCI had better inter-rater reliability for co-morbidity measurement in PD. The CCI was also quicker to score than the CIRS. Furthermore, the CCI was the only scale that was independently predictive of mortality in PD. This is the first study to compare different scales for the measurement of overall co-morbidity burden in PD and these factors suggest that the CCI may be preferable for use in future studies in PD. It is likely that the CCI has better inter-rater reliability because (i) it specifies precisely which diseases are to be included so is less subjective than the other scales, especially the CIRS which includes assessment of disease severity; and (ii) because there is less variation in the scoring than in the other scales.

It is unclear why the other two scales were not predictive of mortality. Only one previous study has published data on the association between baseline co-morbidity and mortality in PD [20], [21]. It found that cardiac co-morbidities (but not other types of co-morbidity) were associated with mortality. One study in PD showed that the overall co-morbidity burden was associated with poorer outcomes in terms of disability and quality of life [13], but no previous study has reported the association between overall co-morbidity burden and mortality in PD or in other parkinsonian disorders. The diseases comprising the CCI were selected according to their association with mortality [9], which may explain why it would have a stronger association with mortality than the other scales, but it is surprising that no independent association was observed with the other scales. It may be that many chronic illnesses included in the other two scales do not have a strong association with mortality which dilutes the effect of those illnesses which are associated with increased mortality. Lack of power is a possible explanation for the lack of evidence of an association between other scales and mortality.

Our finding that co-morbidity burden was strongly associated with mortality in the first period of follow-up, but was not associated in the later period, is novel. This is probably because, as our data shows, early deaths in PD are more likely to be related to other diseases than due to PD itself as PD is not usually rapidly progressive [8]. By contrast, later deaths in PD are more likely to be due to complications of PD itself or co-morbidity which developed after diagnosis. It is therefore important to adjust for baseline co-morbidity in prognostic studies of short-term outcomes, whereas, if long-term outcomes are of more interest, measurement of, and adjustment for, accrued rather than baseline co-morbidity may be more important.

We did not find any association between baseline co-morbidity and the development of functional dependency. No previous studies have investigated the association between co-morbidity and dependency [22], and the only study which has investigated co-morbidity burden as a prognostic factor in terms of disability in general found that it was independently predictive on one measure of disability but not another [13]. It is likely that the burden of co-morbidity is therefore much less important in terms of the development of dependency that factors related to PD itself.

We found no significant difference in overall baseline co-morbidity burden between cases and controls and no difference in the accrual of co-morbidity over time. Two previous studies also showed similar co-morbidity burden in PD and controls [12], [23]. Conversely, one study reported about twice as much co-morbidity in PD than in controls [24], but in that study, the mean age in patients was 69, compared to 45 in controls, and the confounding effect of age was not adjusted for in the analyses. This is the first study to report the accrual of co-morbidity in PD, and to compare this with controls. As dementia is included in the Charlson, and is commoner in PD that in controls, we expected the rise in co-morbidity burden to be greater, but we did not find this, maybe because we analysed data only up to five years, at which time most patients have not developed dementia.

This study has several strengths. Firstly, recruitment of patients had low risk of selection bias. Patients were recruited from the community, were recruited at the time of their diagnosis, and, furthermore, as the cohort was derived from an incident study we attempted to recruit all new patients with PD in the geographical area in the incident period. Additionally, there was a high consent rate to follow-up and very few losses to follow-up. Secondly, control recruitment was community-based and controls were age-sex matched to patients. Thirdly, we had access to comprehensive co-morbidity data (including primary care record summaries) and were able to verify participants’ self-report. Similarly, data on death were comprehensive and accurate. Fourthly, we strove to analyze the data carefully, with adjustment for confounding and for correlation due to repeated measurements, where appropriate, and avoided including too many variables in the models. In terms of the association with prognostic outcomes this study meets the criteria previously recommended for prognostic factors studies [25].

Nevertheless, there are some limitations to this study. While there was adequate sample size for comparisons between cases and controls, there was limited power for identification of weak associations with outcomes. Selection bias in control recruitment is possible as the consent rate amongst approached controls was 44%. While not an unusually low consent rate, it has the potential to introduce selection bias. We previously investigated this possibility and found that consenting controls were broadly similar to those approached who did not consent, but that the consenters scored slightly worse on some health-related metrics [26]. In the five years over which the change in mortality was studied, the risk of death was higher in patients than in controls. Although a linear mixed model can handle missing data, assuming it is missing at random, the differential rate of death may introduce bias [27].

These findings are important for future prognostic studies in PD. Co-morbidity is an independent prognostic factor and may therefore be a confounder in the relationship between other prognostic factors and outcome in PD. Further work is needed to examine the relationship between co-morbidity burden and other prognostic factors. For instance, aspects of social support may be important influences on the relationship between co-morbidity and outcome. These data suggest a very weak effect of co-morbidity burden on dependency, but this may be due to chance and a larger study with more power may be able to estimate this more precisely. Co-morbidity burden should also be included in prognostic models of outcome in PD, which may be useful in predicting individual outcomes or in clinical research for case-mix correction or for stratifying randomization in clinical trials.

## Financial disclosures/conflicts of interest

Dr Macleod was funded by a Clinical Academic Fellowship from the Chief Scientist Office of the Scottish Government and by NHS Grampian endowments and received grant funding from Parkinson’s UK relating to this research. Miss Goddard reports no financial disclosures relating to the research covered in the manuscript. Dr Counsell received grant funding from Parkinson’s UK, National Institute for Health Research, the Scottish Chief Scientist Office, the BMA Doris Hillier award, RS Macdonald Trust, the BUPA Foundation, NHS Grampian endowments and SPRING relating to this research. We declare we have no conflicts of interest.

## Financial support

This study was funded by Parkinson’s UK, the Scottish Chief Scientist Office, NHS Grampian endowments, the BMA Doris Hillier award, RS Macdonald Trust, the BUPA Foundation, and SPRING. The funders had no involvement in the study.

## Authors’ roles

Dr Macleod conceived the study, gathered data, analysed data, and wrote the first draft of the paper. Miss Goddard gathered data, analysed data, and wrote the first draft of the paper. Dr Counsell conceived and recruited the PINE study cohort, conceived this study, gathered data, reviewed the analyses, and reviewed and critiqued the paper.

## Figures and Tables

**Supplementary Fig:**
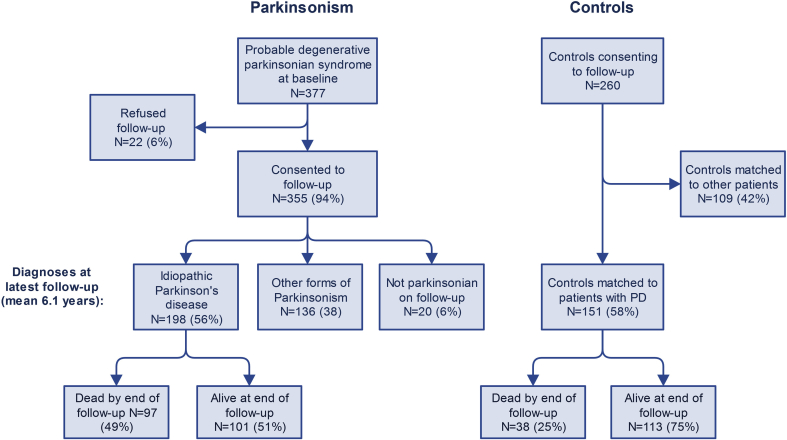
Flowchart of patients and controls in the PINE study.

**Table 1:** Baseline characteristics of patients with Parkinson’s disease and matched controls.

Baseline variable	Parkinson’s disease (N = 198)	Controls (N = 151)
Mean age (SD)	72.5 (10.4)	73.6 (9.8)
Male sex, n (%)	119 (60)	96 (64)
Ever smoked, n (%)	89 (45)	95 (63)
UPDRS Motor Score, mean (SD)	25.1 (11.6)	3.3 (3.8)^b^
DepCat Score^a^		
1–2	106 (54)	76 (50)
3–4	54 (27)	40 (26)
5–6	38 (19)	35 (23)
Presence of any tremor, n (%)	170 (86)	19 (13)^c^
SE-ADL	90 (80–95)	100 (95–100)^d^

a 1 most affluent, 6 most deprived.

b 6 missing data.

c 4 missing data.

d 6 missing data.

**Table 2:** Reliability and associations with prognostic outcomes of three co-morbidity scales in Parkinson’s disease.

Scale	Reliability		Association with mortality^b^		Association with dependency^c^	
Type of reliability	ICC (95% CI)^a^	Crude hazard ratio (95% CI)	Adjusted hazard ratio (95% CI)	Crude hazard ratio (95% CI)	Adjusted hazard ratio (95% CI)
Charlson	Intra-rater	0.98 (0.96–0.99)	1.30 (1.15–1.49)	First four years: 1.27 (1.08–1.49)^d^ After four years: 0.84 (0.64–1.10)^d^	1.25 (1.09–1.44)	1.10 (0.95–1.26)^d^
Inter-rater	0.96 (0.91–0.99)

CIRS	Intra-rater	0.99 (0.95–1.00)	1.07 (1.01–1.12)	1.00 (0.95–1.06)^e^	1.10 (1.05–1.16)	1.03 (0.98–1.09)^e^
Inter-rater	0.40 (0.10–0.74)

Disease Count	Intra-rater	0.96 (0.91–0.99)	1.10 (1.00–1.20)	0.98 (0.88–1.08)^d^	1.18 (1.08–1.29)	1.05 (0.95–1.16)^d^
Inter-rater	0.62 (0.11–0.85)

a N = 20 for each reliability comparison.

b N = 198 for analyses of association with mortality.

c N = 162 for analyses of association with dependency.

d Adjusted for age, sex, deprivation score, smoking status, and UPDRS motor score.

e Adjusted for age, sex, deprivation score, and UPDRS motor score. Abbreviations: ICC = intraclass correlation co-efficient; CI = confidence interval; CIRS = cumulative illness rating scale.
